# Psychosocial stress factors in families with preterm infants during the Covid-19 pandemic: a cross-sectional study

**DOI:** 10.1186/s13034-025-00890-9

**Published:** 2025-04-04

**Authors:** Alina Fendel, Tamara Fuschlberger, Anna Friedmann, Ina Nehring, Marcus Krüger, Volker Mall, Verena Kraus

**Affiliations:** 1 Division of Neonatology and Paediatric Intensive Care Medicine, Perinatal Centre, München Klinik Harlaching, Munich, Germany; 2 TUM School of Medicine and Health, Department of Social Paediatrics, Technical University of Munich, Munich, Germany; 3 TUM School of Medicine and Health, Department of Social Paediatrics, Department of Paediatrics, Paediatric Neurology, Technical University of Munich, Germany, Germany; 4 German Centre for Child and Adolescent Health (DZKJ), partner site Munich, Munich, Germany

**Keywords:** Covid-19 pandemic, Pandemic burden, Preterm infants, Follow up care

## Abstract

**Background:**

The Covid-19 pandemic restrictions severely impacted parents’ and children’s mental and physical health. Families with pre-existing stress factors may have been particularly affected. Parental stress following premature birth is well acknowledged. The addition of the pandemic restrictions to stressors related to premature birth may constitute an especially high-risk factor for family stress and thereby neuropsychological development. Accessibility to special preterm follow-up care programs is important for neuropsychological development and faced additional relevance in the face of pandemic related stressors. We studied the hypothesis that families with preterm born infants were particularly adversely affected by the pandemic-related restrictions in comparison to families with term born infants. Specific stress factors were explored as well as the accessibility of support programs.

**Methods:**

In this cross-sectional study, families were recruited in a neonatology unit with the highest level of care according to German regulations. A questionnaire on perceived pandemic stress factors including amongst others: social contacts, family support, intrafamilial conflicts, leisure activities, and family planning was completed by 101 parents of prematurely born infants while pandemic related restrictions were still in place. We distinguished preterm infants with low gestational age and birth weight from other preterm infants and term born infants. T-tests, Chi-Square tests, Kruskal-Wallis tests, and binary logistic regression analysis were applied to compare the subgroups.

**Results:**

The stress levels resulting from restricted social contacts, family conflicts and accessibility to family support services were significantly higher in families with term-born infants. The accessibility of targeted follow-up care programs for preterm infants was significantly lower for moderate and late preterm infants. The pandemic has had an influence on the decision of parents to have more children in both groups.

**Conclusion:**

Families with preterm infants were less stressed by the pandemic than those with term infants. Targeted follow-up care focused on high-risk groups and left moderate and late preterm infants without medical check-ups. This reflects the general focus in society on high-risk populations during the pandemic. The pandemic had an influence on family planning in both groups.

**Supplementary Information:**

The online version contains supplementary material available at 10.1186/s13034-025-00890-9.

## Background

The Covid-19 pandemic caused enormous alterations in all areas of life. Imposed measures to decrease infection rates required contact restrictions, economic shutdown [1, 2] and resulted in isolation, uncertainty, and high stress levels for many individuals. These disruptions of everyday life, paired with fear of infection of themselves or loved ones put the physical and emotional well-being of children and their families at risk in numerous ways [3–5]. Parents were confronted with additional responsibilities such as daycare, school-related tasks, and compensation for their children’s lack of social contact. This often came along with a duty to work from home and job/economic instability [4, 6].

Families with younger children were particularly burdened by the pandemic [4, 7], as were families with children with heightened care requirements due to pre-existing stress factors such as disabilities, neurodevelopmental disorders, or mental health conditions [8–12].

Preterm infants, i.e. born before 37 weeks of pregnancy, are also considered as such and constitute a risk group for neuropsychological consequences such as cognitive, social, and emotional disorders [13–15]. In this context, parents experience high stress levels, given that they have often faced complicated pregnancies, health issues of mother and infant, and stressful perinatal periods [16, 17]. Parents of children with health challenges such as preterm infants are at a higher risk for mental health problems themselves [18]. This, in turn, is linked to poorer neurodevelopmental outcomes for their children [19]. Mothers of very preterm born infants, i.e. born before 32 weeks of pregnancy, showed higher levels of depression during the Covid-19 pandemic than during the pre-pandemic period [20]. During the pandemic, contact restrictions on neonatal intensive care units (NICU) were widespread. Some institutions had a “no visitors policy” which included the parents, in other hospitals parents were allowed to visit for a limited time and only one parent per day. This has caused great stress and anxiety in parents [21–23]. Physical touch is central to intimate relationships and a protective factor against anxiety, stress and depression. The greater the lack of intimate touch, the more prominent the symptoms of anxiety and loneliness appear [21]. Not being able to touch their own child is positively correlated with anxiety, helplessness and depression amongst parents (*n* = 20 parents of 10 babies) [22].

Protective behaviour in mothers with preterm infants was significantly elevated when compared to term infants. One study examined very low birth weight infants with contact restrictions in the NICU in two German speaking centres. The imposed reduction in bonding resulted in higher parental anxiety and stress [24]. Another question-based cohort study across Germany showed self-imposed restrictions in female caregivers of preterm infants due to fear of Covid-19. This led to enhanced protective behaviour affecting several aspects. These included therapy attendance for the premature child, increased hygiene measures such as hand washing and disinfection of contact surfaces, increased awareness of healthy eating and a turn to alternative medicine [25].

The negative impact of contact restrictions was especially evident for mothers with neonates at the neonatal intensive care unit, as they experienced less social support as a consequence of the pandemic [26, 27]. A higher parental stress level has a negative influence on internalizing and externalizing behaviour– a part of the emotional and social development of children [28]. Internalizing behaviour describes a pattern of emotional and behavioural responses directed to oneself and includes depressive or anxiety disorders and somatic complaints. It is associated with adverse long-term effects such as educational problems or substance use [29]. Externalizing behaviour, e.g., aggression and hyperactivity, is directed toward an individual’s environment and poses a major risk factor for later delinquency and violence [30].

However, it remains unclear which specific pandemic-related stress factors contributed to the burden young families felt throughout the Covid-19 pandemic as well as the question, in which way the pandemic influenced parental stress after premature birth.

Accessibility to special preterm follow-up care programs has gained additional relevance in the face of pandemic related stressors. Here, somatic, cognitive and psychosocial consequences of prematurity are examined. Additionally, aftercare focuses on the interaction between the child and parents and the behavioral abnormalities of the children. During lockdown periods, such elective examinations were cancelled and access to neurodevelopmental check-ups was a major concern for preterm infant care worldwide [31]. Scientific research on the accessibility to follow-up care programs is still sparse. Single centres report positive experiences with telemedical follow-up examinations [32] which provide high contentment of parents and caregivers alongside comparable attendance rates to in-person follow-ups [33, 34], despite not yet being able to successfully replace in-person examinations [35]. This, however, was limited to only a few neonatal centres worldwide, and common standardized assessment tools are not validated for telemedical use.

It was observed before the pandemic that individuals postpone childbearing plans in times of uncertainty such as economic crises [36]. Indeed, family planning changed differently across European countries during the pandemic [37]. In countries with stable economic and labour markets, e.g. France and Germany, people were more likely to postpone family planning, whilst in Italy and Spain the proportion to abandon family planning was larger. In this context, we investigated if families with preterm infants changed their family planning in general during the pandemic. The questionnaire did not ask in detail how the plans changed. Analysing family planning is important to estimate the impact of the pandemic on families beyond the pandemic restrictions.

We investigated the psychosocial and medical situation of families with preterm infants during the Covid-19 pandemic. We distinguished very preterm infants below 32 weeks of gestation or a birth weight below 1,500 g from other preterm infants and compared them with families with term-born infants. We hypothesize that families with preterm infants constitute a particularly vulnerable group regarding additional stress factors caused by the pandemic and its restrictions. An increased stress level might be an additional negative influencing factor of the neurodevelopmental outcome of preterm infants.

## Methods

### Study design

The study was conducted as a cross-sectional cohort study and investigates psychosocial stress in families with preterm infants during phases 5–7 of the Covid-19 pandemic (between 07/2021 and 03/2022). This contains the fourth (Variant of Concern, VOC Delta) and the beginning of the fifth wave (VOC Omikron) [38]. The study protocol was approved by the Ethics Committee of the Technical University of Munich.

### Participants

Participants were recruited during neurodevelopmental routine follow-up examinations in a neonatology unit with the highest level of care according to German regulations within a municipal inner-city hospital of maximum care with around 2500 births a year in Munich, Germany. Infants were assessed at the corrected age of 2–3, 6–7, and 12–14 months of age. Examination time points could not exactly correspond to the routine follow up time points because of contact restrictions. As the applied questionnaire is written in German, only parents who sufficiently understand and speak German were eligible to participate.

The cohort was divided into a very preterm group who were born below 32 weeks of gestational age and/or with a birthweight below 1,500 g (*n* = 49) and a moderate/late preterm group comprising all preterm infants beyond these cut offs (*n* = 52). A comparison was drawn to an existing data set of families with term infants (*n* = 94) which were assessed during the same time.

We received completed questionnaires from 101 parents of preterm infants (see Table 1 and supplementary tables for details).

**Table 1:** Sample characteristics of the children

Variable	Frequency			
	Very preterm infants: < 1500 g and/or < 32 wg (*n* = 49)	Moderate/late preterm infants: > 1500 g and > 32 wg (*n* = 52)	Term infants (*n* = 94)	Total (*n* = 101)
Sex: Male (%)	29 (59.2)	26 (50.0)	54 (57.4)	55 (54.5)
Corrected age in months (± SD)	6.94 (± 3.86)	5.40 (± 2,97)	9.23 (± 1.32)	6.14 (± 3.51)
Weeks of gestation (± SD)	29.12 (± 2.28)	34.50 (± 1.45)	39.80 (± 1.39)	31.89 (± 3.26)
Birth weight** (gram) (± SD)	1,132.24 (± 324.30)	2,270.67 (± 439.49)	3,351.33 (± 408.02)	1,718.37 (± 689.89)
Attending nursery (%)	2 (4.1)	2 (3.8)	2 (2.1)	6 (6.0)
Siblings: yes (%)	28 (57.1)	28 (53.8)	52 (55.4)	56 (55.4)
One sibling (%)	20 (40.8)	19 (36.5)	34 (36.2)	39 (38.6)
Two siblings (%)	8 (16.3)	7 (13.5)	17 (18.1)	15 (14.9)
Three siblings (%)	0 (0.0)	2 (3.8)	1 (1.1)	2 (2.0)
Multiple Births* (%)	22 (44.9)	20 (38.5)	3 (3.2)	42 (41.6)
Twins (%)	13 (12.9)	19 (18.8)	unknown	32 (31.7)
Triplets (%)	3 (3.0)	0 (0)		3 (3.0)

* 3 twin pairs and 1 triplet were examined twice but counted as one visit in our study

### Measures

We used standardized questionnaires, asking for sociodemographic characteristics including date of birth, week of gestation and birth weight, country of origin, degree of parental education, number of siblings, and additional psychosocial stress factors as well as perceived pandemic burden. Pandemic burden was defined as stress and personal affection caused by pandemic related restrictions (see description of the questionnaire below for details). The parental pandemic burden correlates moderately with the German version of the Parenting Stress Index, PSI, total score (Eltern-Belastungs-Inventar, EBI, ρ = 0.265) [7, 39].

Slightly altered, we used a self-constructed and standardized questionnaire which was part of the CoronabaBY study dealing with psychosocial stress factors in families during the pandemic phases 2 and 3 throughout Bavaria [7]. The questionnaire was not independently validated, as there were no previously validated instruments available at the time of its use to measure pandemic burden in parents for comparison. However, it has been utilized in various studies, demonstrating correlations with other stress measures (PSI, as mentioned above).

We added the following questions targeting issues relevant for families with preterm born infants having experienced longer periods of hospitalization: “Has the pandemic influenced your decision to have a baby?”, “Did you feel restricted before and after the birth of your child (regarding cancelled baby and mother groups, isolation in the hospital, restricted presence of your partner and/or a closely related person in the hospital)?”, “How affected have you been by the fear of infection with Covid-19 in the hospital?”, “Have you been able to attend targeted follow-up examinations and programs for preterm born infants?”.

### Pandemic related restrictions and perceived pandemic burden

The self-administered questionnaire comprises a total of 37 questions including questions on restrictions of social contacts, family support, leisure activities, and possible increased family conflicts. Fifteen Likert-scaled questions (5-point scale with 1 = “not stressful at all” up to 5 = “very stressful”) were analysed for this study. The perceived pandemic burden was assessed with the questions: “Taken together what do you think: how stressful is/was the Covid-19 pandemic for you (please think of the measurements such as social distancing, but also your personal experiences, related worries, etc.)?” and, for the infants, “How stressful is/was the Covid-19 pandemic for your child?”.

The questionnaire distinguishes between different timepoints: parents were asked to answer the questions for the time of the interview (TI) and for the time of the strongest restrictions.

### Statistical analysis

Distributions of frequencies were calculated in the total sample, distinguishing between the very preterm (< 1500 g birth weight and/or < 32 week of gestation) and the moderate/late preterm group (> 1500 g birth weight and/or > 32 week of gestation). We performed non-parametric analyses with the Kruskal-Wallis test as the Levene test showed no equality of variance, to compare all groups, families with very preterm with moderate/late preterm infants, and families with term infants. Unpaired t-tests were performed to compare means of the whole sample of preterm infants to term born infants and to compare the two groups of preterm infants, respectively. With the Chi-Square test and its corresponding effect sizes (Phi coefficient φ) we detected associations between the groups and single pandemic related restrictions/changes/burden. For both the Chi-Square test and binary logistic regression models, pandemic-related restrictions/changes and perceived pandemic burden were dichotomized into high/very high (point 4 and 5 on 5-point Likert-scale) versus low perceived restrictions (points 1–3), respectively into stressful/very stressful (point 4 and 5 on 5-point Likert-scale) compared to less stressful (points 1–3). Binary logistic regression analyses were conducted with the preterm group, age, gender, sibling status, and level of education (status high and low).

The calculation was done in SPSS, Version 29.0.0.0. P-values < 0.05 were considered significant.

## Results

The three groups very preterm infants, moderate/late preterm infants, and term-born infants have a male proportion of 59.2%, 50.9%, and 57.4%, respectively. The mean gestational age in weeks was 29.12 (±2.2), 34.40 (±1.46), and 39.80 (±1.39), and birth weight in grams 1,142 (±318), 2,261 (±440), and 3,351 (±408), respectively.

In each group, two participants attended nursery care. The proportions of children with siblings are 57.1%, 53.8%, and 55.4%, respectively.

For multiple births, the rates are 44.9%, 38.5%, and 3.2%, with term infants differing from the preterm groups.

For further details, see Table 1.

### Pandemic burden in families with preterm children

Most parents of preterm-born infants perceived the Covid-19 pandemic with its restrictions as overall stressful or very stressful (61.4%). The parents’ report of their children’s pandemic burden was considerably lower with 8.3%.

At the time of the strongest restrictions, 68.3% of the parents felt stressed or very stressed due to the restriction of their social contacts; in contrast, 17.8% felt stressed or very stressed at TI. About 46% of the children were reported to have suffered/suffered very much under restricted social contacts at the time of the greatest restrictions compared to 19% at TI.

Fear of Covid-19 infections of themselves, their child, or a closely related person was true for 68.3% at the time of the strongest restrictions compared to 38% at TI. Whereas the fear of infection in the hospital was lower with 26.4% and 19.4% at these time points.

Restricted general family support services were problematic for 55.4% of the parents. Restrictions particularly in the peripartal period with regards to antenatal classes, isolation in the hospital, and presence of the partner during and after giving birth were a problem for 65.2%. Targeted follow-up care for preterm-born infants took place for more than half of the families, with 56.5%. In these cases, the offered programs met 72.2% of the parents’ need. Not being able to participate in special preterm support services, however, was a difficulty for 37.1% of the parents at the time of the greatest restrictions and decreased to 30.6% at TI. Within the subgroups of the preterm-born infants, a significant difference exists in the accessibility of targeted follow-up care between very preterm and moderate/late preterm infants (p = < 0.001), with the latter not having sufficient access (Table 6). 62.6% of the families did not suffer from changes in childcare situation, 18.2% found themselves restricted in this aspect.

Increased family conflicts of any kind were reported in 11.9% at the time of the strongest restrictions and 5% (TI) respectively. The questionnaire did not ask in detail what types of family conflicts occurred or which family members were involved.

The pandemic activity exerted influence on parents’ decision to have a baby in 8.4% and 2.8% respectively (no statement can be made regarding in which direction as this was not questioned).

For all perceived restrictions and changes in living conditions related to the pandemic, see Tables 2 and 3.

**Table 2:** Perceived Covid 19-restrictions (Preterm/Term)

Kind of restriction	% No or almost no perceived restrictions (1 + 2)		% High or very high level of perceived restrictions (4 + 5)	
	Preterm	Term	Preterm	Term
Parent social contacts, strongest restrictions	25.7	13.8	68.3	77.7
- Time of the interview	68.3	26.9	17.8	53.7
Child social contacts, strongest restrictions	35.0	26.9	46.0	53.7
- Time of the interview	62.0	51.6	19.0	28.6
Family support services, strongest restrictions	25.7	17.2	55.4	60.3
- Time of the interview	40.6	13.8	25.6	77.7
Changes in childcare situation, strongest restrictions	62.6	51.7	18.2	28.6
- Time of the interview	80.8	57.4	7.0	24.5

	Not at all or little stressful (1 + 2)		Stressful or very stressful (1 + 2)	
Increased family conflicts, strongest restrictions	72.3	57.4	11.9	23.5
- Time of the interview	87.1	80.6	5.0	5.4
Worries about COVID-infection, strongest restrictions	19.8	21.2	68.3	62.8
- Time of the interview	33.0	40.4	38.0	40.4
Overall perceived pandemic burden				
Parent	10.9	11.7	61.4	60.7
Child (parent report)	75.0	75.1	8.3	11.9

**Table 3:** Targeted questions for families with preterm born infants (very preterm vs. moderate/late preterm)

Kind of restriction	% No or almost no perceived restrictions (1 + 2)		% High or very high level of perceived restrictions (4 + 5)	
	Very preterm	Moderate/late preterm	Very preterm	Moderate/late preterm
Impact on decision to have a child, strongest restrictions	75.9	87.8	13.8	4.9
- Time of the interview	82.1	85.0	3.6	2.5
Restrictions during peripartal period	3.6	24.4	71.5	63.4
Worries about COVID-infection in the hospital, strongest restrictions in power	64.3	46.3	17.8	36.6
- Time of the interview	65.5	53.7	17.2	24.4
Preterm targeted support programs	44.8	36.8	34.5	44.8
Were you able to attend targeted follow-up care for preterm infants? (Yes)	62.1	52.6		
If yes, were your needs satisfied? (Yes)	79.2	63.6		

### Pandemic burden in families with term as compared to preterm infants

The pandemic burden for the parents of term infants was stressful or very stressful for 60.7% and so comparable to preterm children (61.4%). Reported children’s burden was 11.9% in term infant families and 8.3% in preterm infant families. The restricted social contacts of parents and their children were, at the time of the greatest restrictions, a major problem for 77.7% (term) and 53.7% (preterm) of the participants, respectively, and 53.7% (term) and 28.6% (preterm) at the time of the interview.

In the t-test (Table 5), the mean values of the perception of restricted social contacts while the strongest restrictions were in place differ significantly between the preterm and the term-born group. Parents of term born infants felt more restricted compared to parents with preterm infants (*p* = 0.009). Moreover, the social contacts of the children were perceived as being more restricted for the term-born infants as compared to preterm-born (t-test: *p* = 0.032). The Kruskal-Wallis test confirms the significant difference here between term and very preterm infants. Both aspects do not show significant differences compared with the Chi-Square test (dichotomized Likert Scale, Table 4).

**Table 4:** Proportions of perceived high/very high pandemic-related restrictions calculated by Chi square test

Kind of restriction/change^1^	Preterm (%/*n*)	Term (%/*n*)	Statistical significance (*p* < 0,05)	Effect Size Cohen’s φ^2^
Family support services Time of the interview	25.6/18	77.7/36	0,011	0,184
Increased family conflicts Time of the strongest restrictions	11.9/12	23.5/23	0,022	0,164

^1^ = Based on responding options 4 and 5 on a 5-point-Likert scale

^2^ = Effect size φ: < 0.01 indicates a small effect, 0.3 a medium effect and 0.5 a large effect

**Table 5:** T-test, preterm vs. term infants

	Mean (± SD) Preterm Term	T	Degree of freedom	*p*-value, bilateral	Cohen’s d
Family support programs, Time of the Interview	2.86 (± 1.428) 2.49 (± 1.065)	−2.071	184.395	0.040	−0.294
Parent social contacts, strongest restrictions	3.69 (± 1.412) 4.13 (± 1.119)	2.390	188.288	0.009	0.3340
Child social contacts, strongest restrictions	3.10 (± 1.554) 3.51 (± 1.475)	1.866	191	0.032	0.269
Family conflicts, strongest restrictions	1.88 (± 1.070) 2.32 (± 1.272)	2.592	182.340	0.010	0.374

Family support services were missed by 60.7% of parents of term infants during the time of the greatest restrictions, even more at TI with 77.0%. The accessibility of family support services was missed more by parents of term-born infants (t-test: *p* = 0.040; Chi-Square test: *p* = 0,011, Tables 4 and 5). Increased family conflicts occurred in 23.5% of the families at the time of the greatest restrictions, 5.4% at the time of the interview compared to 11.9% and 5% in preterm infants, thus significantly more in families with term born infants (t-test: *p* = 0.010; Chi-Square test: *p* = 0,022, Tables 4 and 5). A significant difference regarding family conflicts was especially seen in the Kruskal-Wallis test between families of term infants compared to very preterm infants (*p* = 0,040).

More significant differences were found using the t-test between families with term infants and those with very preterm infants. This was the case for the restricted infants’ social contacts (*p* = 0.030) and for increased family conflicts (*p* = 0.040). In both aspects, parents of term children describe a heavier burden. There were no significant differences between moderate/late preterm infant families and the other subgroups.

For all perceived restrictions, see Tables 2 and 3, the comparison of the families’ burden is shown in Tables 4, 5 and 6.

**Table 6:** T-test, very preterm vs. moderate/late preterm infants

	Mean (± SD) very preterm vs. moderate/late preterm	T	Degree of freedom	*p*-value, bilateral	Cohen’s d
Were you able to attend targeted follow-up care for preterm infants*? (Yes/No)	0.83 (± 0.384)/ 0.38 (± 0.490)	4.295	66.541	< 0.001	1.008

### Sociodemographic characteristics predictive for the pandemic burden

A binary logistic regression model (Table 7) was performed to determine the effect of sociodemographic characteristics on predicting the parental pandemic burden. The model was statistically significant, χ²(10) = 18.361, p 0.049, with Nagelkerke’s R^2^ = 0.228. The one variable contributing significantly to predicting the pandemic burden was the corrected age of the child; the older the child, the higher the burden (*p* = 0.004, OR = 1,247, 95%-CI [1.073;1.450]). The remaining variables did not show significant influence on the parental pandemic burden in the model.

**Table 7:** Binary logistic regression model predictive for the parental pandemic burden

Variable	Regression coefficient B	SE	df	*p*-value	OR	95%-CI	
						Lower Bound	Upper Bound
Age	0.221	0.077	1	0.004	1.247	1.073	1.450

## Discussion

In our study on families with preterm infants, we measured the burden of families with preterm infants during the potentially stressful life event of the Covid-19 pandemic as compared to families with term infants.

Our results show that families with preterm infants felt less restricted during the Covid-19 pandemic compared to those with term infants. This disproves our hypothesis, that the pandemic acts as an additional stress factor to the inherent burden of premature birth. Moreover, the stress levels in families both with preterm infants as well as with term infants do not correlate with the degree of the pandemic activity. This is at first sight startling as having infants with a high risk of neuropsychological deficits constitutes a stress factor for the entire family [16, 17, 20].

Our finding that families are more burdened the older the children are, has been proven for toddlers, school age children and adolescents before [40–45]. Stress in these cases results of fulfilling extra-family demands within the family context, especially for school, and day-care responsibilities [40, 43]. Symptoms of psychosocial stress in families with infants and toddlers were shown to have remained and partially increased over the pandemic period, independently from pandemic events [42].

In term infants, the family might be mentally one step ahead in reaching an ordinary routine for baby. Families with term born children may focus, however, on the return to daily routines including nursery care, grandparents’ support, and return to work [8].

It has been assumed previously that complex clinical histories can shift demands and standards towards well-being [46, 47]. Self-imposed social restrictions may include social distancing because of the child’s vulnerability for infections and extended parental leave periods to account for the special needs of the premature baby and do not correlate to perceived parental stress due to daily problems such as eating, crying and sleeping [7]. In preterm infants the stress caused by the constant fear of losing their child might outweigh other stress factors and so be the common wish of both parents. This is consistent with a study on families with preterm babies on the NICU, where no difference was found between the pre-Covid and the Covid phase [20]. The focus of both parents on the preterm baby may also account for less family conflict, which we observed. Family conflict is a major factor in the negative effects of the pandemic and constitutes one of the risk factors for impaired socioemotional development in children [41, 43, 48]. The fact that conflict arose less frequently in families with preterm infants during the pandemic might account for a protective factor concerning neurodevelopment.

Our NICU maintained an open visitation policy, provided that strict hygiene protocols, including regular COVID-19 testing, were adhered to. As a result, parents were granted continuous access to the NICU, and practices such as kangaroo care and breastfeeding were actively promoted, with the restrictions of mandatory mask-wearing at all times and a maximum of two visitors per patient room at the same time. This approach may have served as a protective factor in preserving the parent-infant bond. Given that parental access remained largely unrestricted compared to pre-pandemic conditions, we did not include the aspect of tactile deprivation in our questionnaire assessing parental well-being.

Concerning neuropsychological deficits due to prematurity standardized postnatal care programs are in place. Eligibility is dependent upon risk factors of prematurity and postnatal complications. Developmental outcome of preterm infants before and after the pandemic up to the age of one year did not differ from pre-pandemic groups [49]. Further studies examine the long-term sequelae in older children (Cherkaoui et al., in preparation).

This corresponds to our findings that accessibility to standardized postnatal care programs was available in the majority of infants at risk. This reflects the pandemic situation with its resource scarcity: medical examinations and treatments were restricted to very few high-risk patients, leaving medium- or low-risk patients without medical care [49, 50]. Therefore, the older the preterm patients were, the less access to special programs was provided and the more burdened the families feel. On a larger scale, this reflects society’s focus on vulnerable groups during the pandemic, taking into account that this might be at the expense of other groups. Additionally, during the first year of life, regular appointments with paediatricians are the norm.

The self-reported result of the interviewed parents is biased, as only those families, who were eligible for postnatal follow-up care, have been able to be assessed. Moreover, demands of these families were satisfied easing pandemic and parental stress by the personal visit to the outpatient clinic. Experiencing social or institutional support serves as a protective factor for the well-being in challenging phases of life [51].

However, pandemic stress had, in a substantial percentage of the families, an influence on their family planning. Whether this effect is long-lasting cannot be answered. Regarding the long-term effects of the pandemic restrictions reaching beyond the pandemic phase, the burden remains high despite the return to normal life [7, 42, 52].

Our findings are in line with the results of a study dealing with the psychological burden during the pandemic in female caregivers of preterm versus term-born children up to 18-years [25], who found that the caregivers of term children suffered significantly more from depression symptoms than those of preterm children.

In the future, effects on the development of children might be at stake because of sustained high-stress levels in the families, an important risk factor. Whether the reason for this effect lies with the pandemic itself or on other increasing problems, e.g. war and climate change, remains to be determined [40, 53].

Our study’s limitations include a relatively small sample size and a referral bias, which is inherent to a monocentre study and makes generalizations challenging. The parents were mostly well-educated and spoke German, few families had low socioeconomic status and migration background. This mainly well-situated cohort can inform themselves and has access to specialist information. Studies on families with lower education levels are necessary. Additionally, in the middle of the pandemic, some parents were not willing to take their children to the hospital in fear of an infection and thus refused participation. Therefore, the families taking part were already a selected group which was, generally, less anxious regarding the pandemic. During lockdown periods, elective examinations were prohibited by law, so the timespan for the study was limited and a certain number of possible participants could not take part resulting in a small sample size. A different timespan during the pandemic might have changed the results of the study.

Moreover, we used self-constructed questionnaires because standardized and validated questionnaires containing pandemic specific questions did not yet exist at the beginning of the pandemic. Overall pandemic burden correlates moderately to the PSI. As this questionnaire has been used in other studies of our research group in the meantime, standardization and validation in comparison with other standardized questionnaires can be assumed. Another limitation consists in the missing control group. This is due to inherent pandemic effects.

We estimated the severity of preterm complications using defined risk groups, but did not stratify for additional organic sequelae such as small for gestational age, intraventricular haemorrhage, broncho pulmonal dysplasia, short bowel syndrome because of necrotizing enterocolitis and the length of stay in the NICU. Another limitation is the retrospective nature of the study concerning the time point of the greatest restrictions and also the second time point of the interview, as this was combined with preterm postnatal follow-up care. Parents might, at this moment, feel a relief of stress as their questions reaching far beyond the developmental exam could be attended to.

Further studies are necessary to determine the longevity of the effect of the pandemic burden for families and children belonging to risk groups.

## Conclusion

Our study reveals foremost that parents’ stress levels have risen throughout the pandemic in families with infants up to 12 months of age. The stress level is higher compared to what studies found in families with older children.

It is most striking that more parents of term-born infants were reported to have suffered more severely than those of preterm-born children, especially during times that were not perceived as the most critical phases of the pandemic.

Hence, we reject our hypothesis of this study that families with preterm infants particularly vulnerable to additional stress factors given their existing burden.

Another aspect derives from a regression model that presents the age of the child as a predictor for the parental pandemic burden.

Across all investigated groups, parents perceive their own burden as being higher than that of their children.

It is in specific aspects that the burden was most apparent, foremost parental social contacts, family support services and worries about a Covid-19 infection of the child or a closely related person. Parents of preterm-born infants additionally expressed having had a burdensome time during the peripartal period, when closest attachment figures were not available due to restriction rules.

An important contrast exists in the accessibility of follow-up care for preterm born infants. Our results reflect that, for high-risk patients, medical supply and targeted examinations were maintained.

## Electronic supplementary material

Supplementary Material 1

## Data Availability

No datasets were generated or analysed during the current study.

## Abbreviations

TI: Time of the interview
