# Supplementary material for: Psychosocial stress factors in families with preterm infants during the Covid-19 pandemic: a cross-sectional study
Source: Child Adolesc Psychiatry Ment Health. 2025 Apr 4;19:41. doi: 10.1186/s13034-025-00890-9 (PMC11971739; doi:10.1186/s13034-025-00890-9)
Supplement: Supplementary file 1 — Supplementary Material 1 [file 13034_2025_890_MOESM1_ESM.docx]

**Supplementary Table 1:** Sample Characteristics of the parents

| Parents | Preterm | Term |
| --- | --- | --- |
| Mothers who completed questionnaire (%) | 86 (85.1) | 84 (89.4) |
| Fathers who completed questionnaire (%) | 4 (4.0) | 7 (7.4) |
| Both parents who completed questionnaire (%) | 2 (2.0) | 3 (3.2) |
| Mean age mothers at birth in years (standard deviation) | 33.4 (5.2) | 32.9 (4.9) |
| Mean age fathers at birth in years (standard deviation) | 34.3 (5.7) | 35.3 (5.7) |

**Supplementary Table 2:** Sample preterm infants, Corrected Age

| Age | 2-3 Months | 5-7 Months | 11-12 Months | Total |
| --- | --- | --- | --- | --- |
| N / % | 41 / 40.6 | 36 / 35.6 | 24 / 23.8 | 101 / 100 |
| Male | 25 = 61. 0% | 19 = 52.8 % | 11 = 45.8 % | 55 = 53.9 % |

**Supplementary Table 3:** Sample, Gestational Week

| Gestational Age | Early & Moderate Preterm  < 32 SSW | Late Preterm  32 – <38 SSW |
| --- | --- | --- |
| N / % | 35 / 34.7% | 66 / 65.3% |
| Male | 20 = 57.1 % | 35 = 53.0 % |

**Supplementary Table 4:** Sample preterm infants, Birth Weight

| Birth Weight | ELBW  (= < 1000g) | VLBW  (= >1000, <1500g) | LBW  (= >1500, <2500g) | Others  (= > 2500g) |
| --- | --- | --- | --- | --- |
| N / % | 25 / 24.8% | 20 / 19.8% | 42 / 41.6% | 14 / 13.7% |
| Male | 15 = 60.0 % | 11 = 55.0 % | 20 = 47.6 % | 9 = 64.3 % |

| **Supplementary Table 5:** Questionnaire pandemic burden / constraints items | | |
| --- | --- | --- |
| **Variable** | **Item** | **Answer** |
| Reduced family support services | “How restricted do you currently feel in terms of family support services (e.g. cancellation of baby and children's groups, support services, counseling services, and consultation hours; avoiding courses due to fear of infection)?” | 1 = 'not restricted at all' to 5 = 'very restricted' |
| Restricted social contacts of the parents | "How restricted are your private social contacts currently (e.g., with family, friends, acquaintances)? | 1 = 'not restricted at all' to 5 = 'very restricted' |
| Restricted social contacts of the child | "How restricted are your child's social contacts currently (e.g., with caregivers, friends, educational professionals)?" | 1 = 'not restricted at all' to 5 = 'very restricted' |
| Restricted leisure activities | "How restricted are your leisure activities currently (e.g., leisure and sports opportunities, club life, cultural offerings, vacations and travel)?" | 1 = 'not restricted at all' to 5 = 'very restricted' |
| Increased childcare responsibilities | "Has the (planned) childcare situation for your child changed due to the COVID-19 pandemic, so that you currently have more caregiving efforts (e.g., due to daycare closures, loss of care by grandparents, caregiving at home)? | 1 = 'not at all' to  5 = 'very much' |
| Worries about Covid-19 infections | “Are you currently concerned that you, your child, or someone close to you could get infected?“ | 1 = 'not at all' to  5 = 'very much' |
| Financial burden due to COVID-19 | “Is there currently a financial burden due to the COVID-19 pandemic?” | 1 = 'non' to 4 = 'major' |
| Increased family conflicts | “Are there currently more disputes and conflicts in the family?” | 1 = 'not at all' to  5 = 'very much' |
| Overall pandemic burden | “Taken together, what do you think: how stressful is/was the COVID-19 pandemic for you (please think of measures like social restrictions but also your personal experiences, related worries etc.)?” | 1 = 'not at all stressful' to  5 = 'very stressful' |
| Family planning | “Has the pandemic influenced your decision to have a baby?” | 'Yes' or 'no' |
| Restrictions during peripartal period | “Did you feel restricted before and after the birth of your child (regarding cancelled baby and mother groups, isolation in the hospital, restricted presence of your partner and/or a closely related person in the hospital)?” | 1 = 'not restricted at all' to 5 = 'very restricted' |
| Worries of Covid-19 infection in the hospital | “How affected have you been by the fear of infection with Covid-19 in the hospital?” | 1 = 'not stressful at all' to  5 = 'very stressful' |
| Preterm targeted support programs | “Have you been able to attend targeted follow-up examinations and programs for preterm born infants?” | 'Yes' or 'no' |
| Needs satisfied? | “If you were able to attend targeted follow-up examinations and programs for preterm born infants, were your needs satisfied?” | 'Yes' or 'no' |

 English translation of the questionnaire taken from the CoronabaBY study including additional preterm-targeted questions.
